# Digital transformation in organizational health and safety to mitigate Burnout Syndrome

**DOI:** 10.3389/fpubh.2023.1080620

**Published:** 2023-03-21

**Authors:** María-Isabel Sanchez-Segura, German-Lenin Dugarte-Peña, Fuensanta Medina-Dominguez, Antonio Amescua Seco, Rosa Menchen Viso

**Affiliations:** ^1^Computer Science and Engineering Department, Universidad Carlos III de Madrid, Leganés, Madrid, Spain; ^2^Higher Polytechnic School, Universidad Francisco de Vitoria, Pozuelo de Alarcón, Madrid, Spain; ^3^Instituto Regional de Seguridad y Salud en el Trabajo de la Comunidad de Madrid, Madrid, Spain

**Keywords:** Burnout Syndrome mitigation, digital OHS, smart-OHS, occupational health and safety, digital transformation (DX)

## Abstract

In 2000, the World Health Organization (WHO) identified Burnout Syndrome as an occupational risk factor, affecting an estimated 10% of workers, resulting in lost productivity and increased costs due to sick leave. Some claim that Burnout Syndrome has reached epidemic proportions in workplaces around the world. While signs of burnout are not difficult to identify and palliate, its real impact is not easy to measure, generating a number of risks for companies from possible loss of human talent to decreased productivity and diminished quality of life. Given the complexity of Burnout Syndrome, it must be addressed in a creative, innovative and systematic way; traditional approaches cannot be expected to deliver different results. This paper describes the experience where an innovation challenge was launched to collect creative ideas to identify, prevent or mitigate Burnout Syndrome through the use of technological tools and software. The challenge was endowed with an economic award and its guidelines stated that the proposals must be both creative and feasible from an economic and organizational point of view. A total of twelve creative projects were submitted, including each of them, the analysis, design and management plans, to envision an idea that is feasible and with the appropriate budget, implemented. In this paper, we present a summary of these creative projects and how the IRSST (Instituto Regional de Seguridad y Salud en el Trabajo) experts and leaders in OHS in the Madrid Region (Spain) envision their potential impact on improving the OHS landscape.

## 1. Introduction

Given the challenges posed by the emerging digital society, organizations must evolve and adapt their occupational health and safety policies to guarantee the safety of workers in a dynamic and ever-changing work environment, in which also emerging risks such as Burnout Syndrome have appeared. The global COVID-19 pandemic led to the unexpected and rapid implementation of teleworking, often becoming permanent, forcing workers and organizations to improvise work environments (1), requiring new OHS protocols.

**Figure 1:**
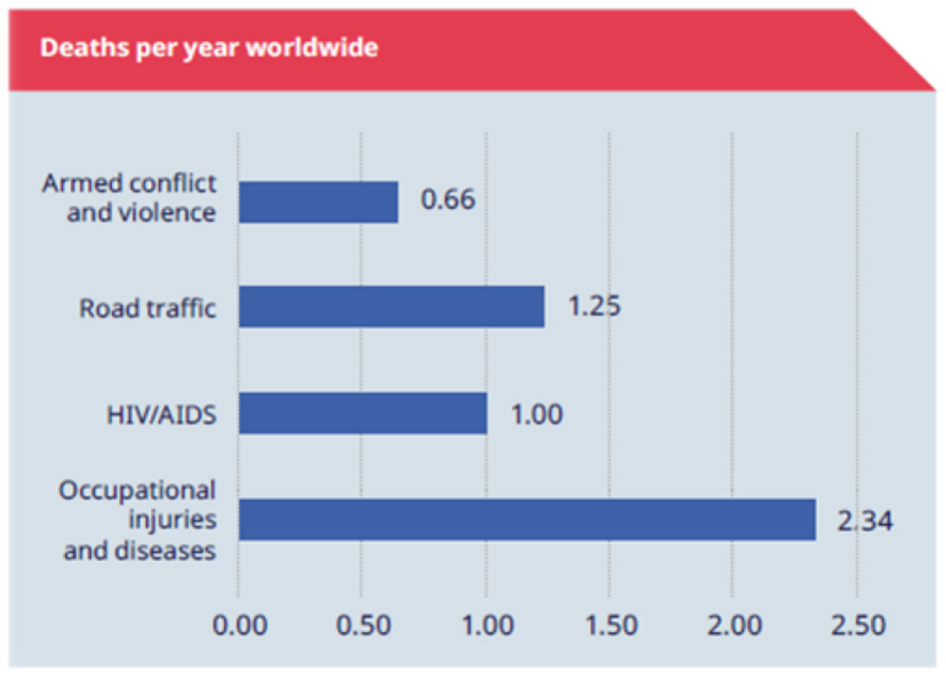
Annual deaths worldwide due to workplace accidents. Source: Laszcz-Davis (3).

One of the priority objectives of the European Union's OHS policy is the implementation of effective occupational health and safety management systems (2), to address the high rates of workplace accidents and work-related illnesses, as shown in Figure 1 (3).

One way of reducing these accident rates is to identify psychosocial mechanisms to mitigate Burnout Syndrome (4), a well-known but largely unaddressed phenomenon due to the difficulty of diagnosis and treatment.

Burnout Syndrome, understood as “*a syndrome conceptualized as resulting from chronic workplace stress that has not been successfully managed. It is characterized by three dimensions:* *(**1**)* *feelings of energy depletion or exhaustion;* *(**2**)* *increased mental distance from one's job, or feelings of negativism or cynicism related to one's job; and* *(**3**)* *a sense of ineffectiveness and lack of accomplishment. Burn-out refers specifically to phenomena in the occupational context and should not be applied to describe experiences in other areas of life*” (5), is probably one of the most pervasive occupational psychopathologies in OHS, and while for non-specialist there is not consensus on its precise definition or diagnosis, it clearly has significant repercussions for both individuals and organizations. The paradox of burnout is that those who are most commonly at risk are those who are most committed and dedicated to their work (6). This is because, according to Mitchell in referring to caregiver burnout (7), “when you feel exhausted and overwhelmed, it will create a change in your attitude from positive and caring to negative and unconcerned.”

This syndrome is not new, and has been the subject of research since 1970 but it wasn't until 2019 that the World Health Organization finally included it in the international classification of diseases (ICD-10) as “*a syndrome conceptualized as a result of chronic work-related stress that has not been successfully managed*”; as of 2022 it is included in the 11th Revision of the International Classification of Diseases (ICD-11) as an occupational phenomenon (8). Tools to assess Burnout Syndrome have existed since 1981 (9), but the panorama has significantly changed since then. According to Moss (4) “*burnout is a serious illness, which is provoking a high increment of suicides, and also it is expected a loss of nearly $1 trillion in productivity globally each year, spend $190 billion in health-care outlays, and 120,000 people will die from burnout in the United States alone*.”

In this paper we will address this occupational phenomenon considering that two variables, “Burnout Syndrome” and “digital technologies,” have grown exponentially in recent years. The question is, given how pervasive digital technologies have become, can these technologies be harnessed to mitigate Burnout Syndrome? There are many informal resources, techniques and strategies for personal self-management and wellbeing, mainly and very little has been done into the real prevention of Burnout at important organizational levels. The motivation for this research is to mitigate the striking lack of formal tools available and addressing this shortcoming.

Believing that new technological solutions can be developed to help to identify and prevent burnout Syndrome, and based on previous research supporting the speeding-up role of technologies to improve OHS (10), in 2022 the IRSST-UC3M Research Chair funded by the Regional Institute for Occupational Health and Safety of the Community of Madrid (https://www.comunidad.madrid/centros/instituto-regional-seguridad-salud-trabajo), launched the “Creativity for Digital Transformation C4DX-OHS” (https://catedrairsst.uc3m.es/en/challenge-c4dx-in-ohs/). The aim is to find creative solutions to Burnout Syndrome through digital transformation that will improve the quality of life of workers. The objective of this work is to analyze the creative projects submitted for the C4DX-OHS challenge and the impact these proposals may have in the field of OHS.

## 2. State of the practice

Given the importance of preventing, identifying and mitigating burnout, it is essential to identify the tools organizations currently have at their disposal. For this, two aspects must be considered.

First, it is essential to identify the tools available, that is, the software solutions companies can acquire to prevent, identify and mitigate burnout among their employees. Secondly, it is also essential to identify the existing frameworks and standards used to assess Burnout Syndrome.

### 2.1. Existing technologies to address Burnout Syndrome (BS)

One well-known OHS tool to treat the BS is Workfit (11), which offers a wellness management program that provides employees access to professional help to improve their wellbeing through activities such as mindfulness therapies, yoga sessions, etc.

There are a number of tools which address (detect) burnout using Artificial Intelligence (AI), for example Erudit AI Inc (12), which offers a SaaS AI tool that works on platforms such as Microsoft Teams, Slack and Zoom. The tool works to detect signs of Burnout by analyzing words, that is the AI analyzes video conferences and meetings, processing the words employees use, their tone of voice as well as their physical appearance. This tool only works with video conferences and calls, and therefore lacks sufficient data to make an accurate overall assessment to detect burnout among employees.

Another example, to detect and treat BS, is RAD AI (13), which helps employees to save time, reduce Burnout and improve patient care. The tool allows mental health experts to detect Burnout using patient data, making it very easy to generate accurate information that can help reduce burnout. While Rad AI and its algorithms provide good insight into what triggers Burnout among patients, it is used in a health care environment, mainly by mental health professionals, and is unsuited to monitoring real-life work environment of an employee.

Cerner's AI-enabled solutions (14) goes beyond traditional processing and focuses on user efficiency to identify problems and inconsistencies within an employee's record, so detecting and preventing BS. This can help to reduce doctors' workloads while maximizing the efficiency and financial solvency of health care systems. This solution is effective as a prevention system, generating a good work environment for employees by managing their work schedules. The data gathered can be very relevant in helping to prevent Burnout.

Nevertheless, the number of effective tools designed to address Burnout Syndrome (BS) are limited. In fact, “*previous attempts at reducing burnout have been initiated by individuals, with no central hierarchy promoting this reduction. By and large, the only source of information to reduce burnout has come from self-help books, articles, and websites*” (15). Most available tools focus their marketing on their ability to change people's minds, helping them recognize their need for support. “*Build your inner peace from the ground up*” states the Sensa website (16), which offers a tailored product “*to guide you through the entire journey to self-care, unlocking scientifically backed information to help you understand yourself better on your own terms*,” with the clear intention to mitigate the risk of Burnout mitigation. This is just one example of the type of tools available.

### 2.2. Existing approaches to burnout prevention, evaluation and assessment

We have analyzed a number of internationally recognized, non-technological approaches to identifying and preventing Burnout Syndrome, discussed in the section below.

#### 2.2.1. Management and personal recommendations

Managerial rules and recommendations abound in every field of organizational management, and employee burnout is no exception. Mitchell, for example, recommends that leaders do three things to maintain employee productivity and prevent burnout (17):

- First, leaders should provide “*the necessary time for their employees' mental health*,” providing time for workers to take care of themselves “*without worrying about work*.” The cost of this approach is open to question since work hours directly impact personnel budgets and company productivity.
- Second, leaders should be “*trustworthy with their subordinates' careers*,” since a trusting relationship “*helps to cut down on feeling like one is unappreciated at work*.” This appears logical since negative feelings are clearly drivers of Burnout Syndrome.
- Third, “*Upper-level management must empower leaders to stop and prevent job burnout*,” thus making employee burnout prevention as strategic goal of the organization.

In terms of self-management, there are many recommendations for improvement. Holiday (18), for example, suggests it is essential to take control of one's life, to be disciplined enough to know when to stop, when to rest, and when to resume work. To illustrate this, the author gives numerous examples from the world of sport, where discipline is the key to long-term success, stating that “*it is important to win the workout, but is more important to win the race*.”

Informal resources, techniques and strategies abound for personal self-management, mainly revolving around the “marketing of wellbeing” and very little scientific or academic research has been done into these approaches. As mentioned, considering the prevalence of Burnout Syndrome there is a striking lack of formal tools available and addressing this shortcoming is the motivation for this research. In the following sub-sections we will analyze two more formal approaches to addressing Burnout Syndrome and briefly analyze the state of corporate strategy in this regard.

#### 2.2.2. Corporate policies

With the digitalization of organizations, the digitalization of human development processes and the management and promotion of talent has also arrived. Specific programs to prevent Burnout so-called “interventions” have arised as a trend at all levels of management both for the economic impact of caring for people and the fact that having satisfied employees is related to positive organizational outcomes. Many programs to manage these interventions and its related challenges have been proposed and compared (19), all of them being beneficial first at a instant level and with long-term effect possibilities through the enhancement with refresher courses.

Examples of this are the programs aimed at investigating how to improve the wellbeing of people, and to do so by improving their working and professional conditions (20). Specifically, it is possible to speak of interventions as an organizational strategy to promote the wellbeing of employees (in this case in the health sector, but extrapolable to other sectors), which is something critical from a professional perspective, due to the impact that Burnout may have on factors such as “*influence quality of care, patient safety, physician turnover, and patient satisfaction*” (21). Also, some works focus on relations and co-dependencies, such as the work of Day et al. (22), who discovered the importance of factors such as job control and support (and supervision) concerning Burnout.

The importance of organizational strategies is such that at a global level formal contributions support this idea: “*The importance of a contextualized health approach with a focus on organizational environments is becoming more strategic than ever due to COVID-19 and the ensuing difficult situation that employees are experiencing worldwide. The prevention of workers' mental health problems is complex and multidimensional, and it is not always possible to protect the person by analyzing personality, psychopathology, and psychiatric syndromes*” (23).

#### 2.2.3. Maslach Burnout Inventory™ (MBI)

The Maslach Burnout Inventory (MBI) (24) measures Burnout as defined by the World Health Organization (WHO) and the International Classification of Diseases ICD-11(5). For many years, the MBI has been widely regarded as the leading measurement tool of Burnout Syndrome, validated by over 35 years of extensive research and mentioned in some 88% of published research into Burnout Syndrome (25).

The Maslach Burnout Inventory (MBI) includes four tools:

- MBI-HSS (MP) Toolkit: for Medical Personnel.
- MBI-HSS Toolkit: for Human Services workers.
- MBI-ES Toolkit: for Educators.
- MBI-GS Toolkit: for General use.

Although the MBI is arguably the most useful and commonly used questionnaire, it “*shows a number of conceptual, technical and practical imperfections*”; This “*is not surprising because the MBI was developed almost 40 years ago for research and not as an assessment tool*” (26).

#### 2.2.4. The Burnout Assessment Tool (BAT)

The Burnout Assessment Tool (BAT) (26) is a new self-reporting questionnaire to measure Burnout, developed as an alternative to the Maslach Burnout Inventory (MBI) which was developed many years ago with research purposes, not even considering to assess BS.

The BAT combines both deductive (theoretical) and inductive (empirical) approaches. In theoretical terms, burnout is understood as a state of mental exhaustion that manifests itself as “*both the inability and unwillingness to spend effort at work*”; the inability to carry out one's work properly because of chronic fatigue: “*I can no longer do my job*”; and an unwillingness to work because of mental distance from one's job: “*I do not want to do my job anymore*.”

Based on in-depth interviews with professionals with long expertise in dealing with burnout among employees, the BAT includes two more critical aspects: emotional and cognitive impairment, that is, the functional capacity to regulate one's emotional and cognitive processes is “*impeded, which manifests itself, for example, in sudden bursts of anger and in poor concentration*.”

The BAT model also considers certain symptoms of psychological stress, psychosomatic complaints and depression. These can be considered as secondary or “atypical” symptoms of Burnout since they occur not only among those suffering Burnout Syndrome but among many others as well. Nevertheless, those suffering from burnout often exhibit these symptoms, and it is important they be taken into consideration.

In developing the Burnout Assessment Tool, over a dozen burnout questionnaires were analyzed, totaling more than 300 items. It was found that “*exhaustion plays a pivotal role in all questionnaires and that mental distance is included in all questionnaires that do not limit burnout to mere exhaustion*.”

## 3. Creativity in digital transformation to mitigate Burnout Syndrome

In this section, we present the challenge launched in 2022 in the form of a call for proposals for digital transformation and the use of new technologies to prevent or mitigate Burnout Syndrome.

### 3.1. Challenge description

In 2022 the posed challenge was to propose a digital transformation project to help address Burnout Syndrome, one of the most significant OHS psychopathologies which requires far greater attention. Proposals must include the use of a software application, hence the acronym C4Dx.

Although there is no consensus on the definition or evaluation of Burnout Syndrome, it is clearly a significant problem for both individuals and organizations. Paradoxically, the most dedicated employees are those who are most at risk; burnout typically afflicts those who are most committed to their work.

University students within the Community of Madrid were called upon to propose creative ideas for digital transformation in the area of OHS, specifically to help mitigate Burnout Syndrome. The challenge was posted at https://catedrairsst.uc3m.es/en/challenge-c4dx-in-ohs/ with a prize of 600 euros for the best creative idea in digital transformation. The proposals must include:

1- Details of the digital transformation proposal: Title, authors, contact email.
2- Description of the digital transformation proposal to mitigate Burnout Syndrome: maximum two pages, Arial 11 font, single spacing.
3- Costs associated with the development of the proposal.
4- Functional and non-functional requirements of the proposal.
5- Analysis and design of the digital transformation proposal.
6- Video presentation in mp4 format (maximum 6 min).

A total of 12 proposals were received and evaluated (more information at https://www.promiseinnovatech.com/challengec4dx?view=article&id=40:c4dx-2022-creativity-for-digital-transformation-in-organizational-health-and-safety&catid=2:uncategorised). A complete list of the proposals and their technological solution is provided in Table 1, below.

**Table 1:** List of proposed creative projects—technological solutions.

**Contribution ID**	**Authors**	**Proposed solution**
Contribution 1	Dahl Aarhus et al. (27)	Virtual personal assistant
Contribution 2	Morata Hontanaya et al. (28)	BonusBreak
Contribution 3	Cebrian Puñuela et al. (29)	Mental health monitor
Contribution 4	Cieslinski et al. (15)	Free time booking app
Contribution 5	Tsai et al. (30)	BreakBuddies
Contribution 6	Madsen et al. (31)	FieldDay
Contribution 7	Alonso (32)	LightUp
Contribution 8	Abella Miravet (33)	Burnout WT
Contribution 9	Izuzquiza Gimeno et al. (34)	TrusTec
Contribution 10	Bernaldo de Quirós de Cal et al. (35)	Bright software
Contribution 11	Bullido Valhermoso et al. (36)	Hubture
Contribution 12	Chiclana et al. (37)	Burnband

The main features of these projects will be described and analyzed in the paragraphs below.

### 3.2. Executive summary of the creative proposals in response to the challenge

#### 3.2.1. Contribution 1

The idea is a Virtual Personal Assistant (VPA), designed both to prevent Burnout among users by encouraging healthy habits and to detect early signs of Burnout by analyzing the user's interaction with the device. The VPA will have a gender neutral name, “Charlie,” and a default gender-neutral voice (27).

This product will be targeted toward companies with employees working from home, who are more prone to lose track of time, to forget to take breaks or to interact with colleagues than their peers working in an office environment surrounded by colleagues. “Charlie” will sync with the user's calendar, fitting as seamlessly as possible to avoid being a distraction or annoyance. This includes muting messages during meetings and suggesting breaks when these are possible for the user. Over time, “Charlie” will collect data about user responses and reactions, tailoring its recommendations and notifications to the users' needs and feelings.

The key aspect of the VPA is to help users manage situations which may put them at risk of burnout. “Charlie” will help the user to set boundaries, while showing understanding and tailoring its notifications, reminders and tips to the user's needs. “Charlie” will monitor the user's mental health on a score of 1–10 through weekly check-ins, simple conversations, analysis of habits and responses to questions throughout the workday. With this data, “Charlie” can create a score of how it perceives the user's mental state and tailor its content accordingly. If the user score is approaching a level that indicates symptoms of Burnout, “Charlie” will send more reminders about breaks, meditation, breathing exercises, home stretching etc. If a user score reaches 2 or 3, “Charlie” will recommend talking to a manager or therapist to take action in dealing with stress.

The product can be personalized by the user. They can customize “Charlie” to have a female or male voice, decide the intervals at which messages and reminders are received, and set the schedule for weekly check-ins with “Charlie.”

##### 3.2.1.1. Originality, contribution, and limitations of C1

The main goal of this Virtual Personal Assistant (VPA) is to prevent Burnout among employees working from home by keeping track of their mental health through weekly check-ins. This data will be used to give personalized notifications and reminders to meet the user's needs. The tool will also synchronize with the user's calendar so that it can customize settings, for example, not giving notifications during meetings, etc. Currently, there are no similar tools on the market although there are alternative solutions that can be used to develop the VPA. Comparing these alternatives and existing technologies, the design team concluded that Deepgram could be an appropriate tool for Automated Speech Recognition, Raspberry Pi as the device and using Google's text-to-speech technology. Overall, the design team believes the Virtual Personal Assistant may be an interesting idea, given there is currently no similar alternative. Considering the prevalence of Burnout Syndrome among employees, particularly those working remotely, this idea could be successful if sufficient funding or support were obtained.

#### 3.2.2. Contribution 2

BonusBreak seeks to create a unique experience that enables each individual user to track their mental wellbeing in the workplace, while also providing incentives at the team level to create a healthy work environment. The main target market is software engineers, where teams work according to repeating cycles, for example, 2-week development sprints for specific tasks within a multi-stage project. On an individual level, users of the app will complete a brief, initial survey, describing their current workstyle, habits for de-stressing, interests and hobbies outside of work and any current causes of stress or anxiety in the workplace. With this data, the app will offer specific routines tailored to each user, including scheduled break reminders and suggested activities during these breaks. These activities will focus on a user's preferred interests and de-stressing routines. The platform will allow users to visualize and keep track of how performing the recommended tasks impacts their workstyle, productivity and overall wellbeing. During their workday, users will receive pings with spontaneous notifications from the app asking them to describe their current feelings. All of this data will be incorporated into the app (28).

##### 3.2.2.1. Originality, contribution, and limitations of C2

BonusBreak aims to simultaneously prevent and to reduce Burnout Syndrome among employees. The main limitation of this project is that it will only work if both the employees and team managers make the effort to combat Burnout by using the app. BonusBreak should provide incentives for employees, offering rewards while also actively combating Burnout, promoting a more collaborative work environment which is also effective in combating burnout, and encouraging employees to make use of the application.

BonusBreak differs from existing tools as it oriented toward both individuals and teams. On the individual level, a point system will offer employees incentives to do tasks and activities that will reduce the likelihood of burnout, for example, taking breaks, giving positive feedback and complimenting coworkers, and receiving bonus points for consistently using the app. On the team level, the app offers a reward system that incentivizes a healthy work-life balance, tracking the amount of time employees spend working during the day, the tasks completed, and giving employees the option of voting on what rewards they would like, for example a social event such as dinner, trips or excursions, or additional holiday time or a monetary reward.

#### 3.2.3. Contribution 3

This solution is based on an evaluation system which uses two different methods to collect information from employees to evaluate their mental health and offer solutions if they are in danger of burnout. The full-coverage method doesn't require the intervention of humans or third parties to work, it encourages employees by displaying in different formats their contribution to the final product and includes a meditation and mindfulness system. The partial-coverage method works by suggesting ways for the user to prevent monotony and stress, prompting them in various ways to stay positive and to take advantage of help from superiors, the human resources team and other professionals if needed, online or in-person. The system uses Artificial Intelligence to monitor employee habits and using a scoring system to identify the risk of employee burnout. When an employee surpasses certain thresholds, a number of possible solutions, both for prevention and mitigation of burnout, will be created (29).

##### 3.2.3.1. Originality, contribution, and limitations of C3

This application is designed to detect and mitigate symptoms of employee burnout. The proposed solution will target prevention over recovery, although some recovery solutions will be provided, given that the system's primary goal is to prevent burnout from appearing. Employees can avoid stress through greater awareness of their breathing and the use of relaxation techniques. One of the features of this proposal is to provide organizations with meditation and mindfulness resources to offer their employees. There is, however, a degree of dependence on third parties to implement this solution.

The designers claim that “*not seeing how the contribution of your work affects the final product, may lead to lack of meaningful tasks*.” Hence, this application includes a feature that shows every employee how important their work is to the goals of the company. Videos and/or messages express the gratitude of the final user, what the final product would look like without the employee's contribution, positive feedback and acknowledgment of the employee's work from managers and supervisors, the degree to which the employee's work has saved time, effort or expense of others on the project and a projected timeframe of the project when everybody contributes their part.

Another key aspect is the mitigation of burnout risk in the event of long periods of demanding work. The software will suggest employees have a break, take a walk, or rehydrate to clear their mind. There is also a two-part check-up system, in-person and online. The online check-up will give pop-up reminders to take a break and includes a journal to record tense situations to help reduce stress. The in-person check-up will encourage employees to contact a mental health professional or take a daily walk with someone at the office.

#### 3.2.4. Contribution 4

The idea is to develop an application/website through which employees can book time off from work, spending time in a rented room where they can play games or practice individual or group meditation. Employees can earn points based on the number of hours worked, meeting deadlines, and keeping high efficiency score, which can be spent at company-approved activities or extra benefits. These break times help minimize employee burnout by allowing them to unwind, de-stress and detox. Company managers can approve worker schedules and request modifications from employees if necessary. This ensures compatibility and coordination between staff and management to delivery long-term wellbeing (15).

##### 3.2.4.1. Originality, contribution, and limitations of C4

At the company level, the programs and resources to reduce employee burnout are limited, consisting mainly of having employees “check in” with minimal perks or benefits. This proposal is innovative in putting the power into the hands of employees, who can decide, with company approval, when to take breaks and choose their rewards.

The proposal allows companies to keep track of employee working hours and weekly objectives, informing employees about their weekly efficiency and, when performance is high, allow them to book relaxation rooms when available.

To implement the system there are shared calendars for relaxation rooms, allowing employees to make reservations *via* email. Names are given to each room to be recognizable to everyone. From the design perspective, it is a good idea that employees are familiar with their rest environments.

Using a hybrid cloud system may be considered, combining the infrastructure of a local server or a private and public cloud. This is used in some companies to address the problem of latency and when control of private data is necessary, this is also a possibility.

Among the many alternatives, a self-automated system with management oversight is best when compared to alternative partial solutions, such as a system where employee rewards are assigned based on the meeting of weekly objectives which may seems arbitrary as weekly objectives may vary widely, whereas a system where reservations are made by email may be inefficient, requiring someone to be responsible for receiving and administering these emails constantly.

In the final proposal, employees who clock up a certain number of hours each week can reserve time off in company facilities for personal rest and relaxation. Employees are empowered to create and manage their own schedules while management can approve or deny these schedules and maintain a degree of oversight. With this approach, the goal is for employees to be happier, more energized and productive.

#### 3.2.5. Contribution 5

This proposal, Break Buddies (30), encourages employees to take breaks while connecting with others by automating decisions and removing barriers. This will make is easier for employees to take breaks during the workday by automatically organizing them together in convenient time slots. Based on each participant's interests and preferences, it provides content in the form of video or audio to facilitate the activity. Having a plan allows participants to get started right away, rather than spending time figuring out what to do. Creating a solution which engages groups can generate added value in two ways. First, it fosters the establishment of social relationships in the workplace, which in turn improves employee mental health and creates a positive work environment, thus reducing burnout. Another benefit of focusing on group activities is that it allows a larger sample size to determine which activities, times, group organizations and other variables work best to reduce employee burnout and produce other positive outcomes.

The target audience for this solution is large organizations with presential office workers, where it can be difficult to engage people individually, especially when certain negative work cultures have taken hold.

The solution functions as follows: Break Buddies will schedule employee breaks automatically with some degree of customizability, integrating with the company work scheduling and meeting room booking systems. It will automatically choose the perfect time to schedule an activity based on the schedules of individual participants. The system will help create groups based on availability, interests, personal invitations, etc. To encourage attendance, groups will be small, customizable to be held more often to encourage frequent breaks. Depending on the activity and group size, the software will automatically select a suitable meeting area. The activities, which may involve viewing or listening to selected content, will be sourced from third parties while users may suggest content or activities to add to the platform.

##### 3.2.5.1. Originality, contribution, and limitations of C5

This proposal consists of a web application that can be easily accessible on employee devices. Each employee will have an account and profile, able to adjust their preferences with certain activities hosted on the platform such as videos, audio, and web games. Users can set up their accounts using their existing organizational credentials to synchronize their schedules and other services. Employees will set up their profile, indicating the activities they enjoy, customize their availability, frequency of activities, group sizes, and invite others to join groups. Users will be informed of the groups or activities they have been added or invited to. They will also be able to choose the activities they wish to join, or indicate their reasons for not joining, thus helping to curate their experience as well as give feedback on the activities to help with future recommendations. Company administrators will be able to see employee trends: identify popular activities and other dynamics and determine which activities help reduce employee burnout. All feedback will be anonymized to ensure the privacy of employees.

The app itself is linked to a web server and database with user authentication details and schedules, able to sync with existing company systems. Logged in users are stored in tokens on the web application. An algorithm will be used to match events and activities to employee schedules, their interests and preferences, and be dynamically updated based on the user response. The web application will have a standard login screen, followed by menus for any of the features they need. Notifications and booked events are displayed prominently to ensure users remember them. Users can set their preferences, indicating what works best for them, making changes to their settings as they become more familiar with the system. For app supported activities (i.e., movies, YouTube videos, certain web games) users can access directly using the app. For non-digital activities (i.e., meditation, exercise, and nature walks), the app will provide helpful information, schedules and instructions. Company administrators will be able to select authorized activities across the entire organization, modify group and room sizes, manage organizational dynamics and receive feedback and user trends.

#### 3.2.6. Contribution 6

FieldDay (31) is an app that, on the first of each month, proposes a game with a particular theme. Every month, employees can play the game once a week, initially every Wednesday to break up the work week, although the day can be changed if necessary. The games are chosen from a series of short, entertaining online games (Wordle, Among Us, Flappy Bird, Doodle Jump, etc.). Throughout the month, the app will present a leaderboard that shows employee rankings within the company, and how the company ranks compared to others. Companies can incentivize employees to play well by offering fun bonuses. The program also offers activities, such as an online escape room, that involve entire work teams which can help build relationships between employees and create a more positive work environment. Finally, some games will be played in pairs, with a different partner every month helping everyone in the company to get to know each other. These games will help improve relationships between employees, decrease stress, and ultimately decrease the risk of employee burnout.

With FieldDay, it is imperative that everybody in the company participates, from interns to the CEO, to ensure bonds and relationships are created both horizontally and vertically within the company. If only a few employees participate, the connections developed between employees will be minimal, defeating the purpose of FieldDay. Managers may believe that FieldDay will decrease productivity, but in fact the opposite is the case. Dedicating short periods of time to build social connections within the company and decrease stress will increase productivity. Ultimately, FieldDay is an innovative, satisfying and entertaining way for companies to address employee burnout.

##### 3.2.6.1. Originality, contribution, and limitations of C6

This solution is based on Locke and Latham's Theory of Goal Setting (38), which supposes that setting and accomplishing goals leads to satisfaction and motivation. It may also lead to the opposite: frustration, lower productivity, and/or motivation. Given that these negative emotions are the principal drivers of Burnout Syndrome, it is important to provide employees with a fun and competitive workplace environment, setting alternative goals for employees to focus on and experience empowering results. FieldDay specifically addresses the hierarchy of employee needs, starting with building strong social connections and loyalty toward the company. This innovative product will help develop a sense of shared purpose within the company and among employees, creating a positive work environment and decreasing employee stress, reducing the risk of employee burnout.

#### 3.2.7. Contribution 7

The Light-Up application (32) collects data using a wristband and other data entered manually by the user, determining whether the combination of parameters is abnormal non-limiting or abnormal limiting and executing one function or another in each case. The application also offers users the possibility to sign up for various activities organized by the company, offer tips on burnout prevention and, depending on the activities of the user, will recommend activities that match their interests. The Light-Up Watch application is installed on a smart bracelet from an external supplier, synchronized with the user's account and measures various parameters.

##### 3.2.7.1. Originality, contribution, and limitations of C7

Light-Up is designed to promote a healthy lifestyle among company employees, helping them to find the right work/life balance.

The designers did not find “*any company specialized in preventing and treating burnout*” in Spain although companies are now gradually increasing the number and variety of activities to reduce stress in the workplace. More action is necessary in this area given that statistics show that Spain is one of the European countries with the highest levels of work-related stress, affecting up to 60% of employees (39). This underscores the viability of this project and provides further incentives to reduce occupational stress.

There are a number of limitations to this proposal: the wristbands are obtained from external suppliers and the maintenance and repair of wristbands is not included in the project, this being the responsibility of suppliers. The project also does not include the installation of the application on user devices, this also being outsourced to suppliers as an additional cost.

#### 3.2.8. Contribution 8

This project is for the design and development of burnout WT (33), a software for the detection and prevention of burnout among Telefonica employees, offering solutions to detect and address this problem. The application will be added to Telefónica's existing management platform, and will thus have access to the necessary employee information, such as e-mails, credentials, work schedules, clock-in management, etc.

Every week, the application will collect employee data related to:

- BAT (Burnout Assessment Tool) questionnaire (26).
- Piezoelectric and distance sensors.
- Communications sent by e-mail and other company platforms.
- Check-in/out time recording.
- SmartWatch sensors.

The AI developed by WellTech, called WELLIA, will interpret the data and generate two types of reports:

- The first contains detailed and personal information of the employee. This report will be confidential, available only to the employee and the company doctor.
- The second consists of group reports at the departmental and corporate level submitted to the Human Resources department to evaluate the general status of employees (this will not include individualized data).

Both of these reports include a series of recommendations to address situations of stress and potential burnout. In this case information can be personalized.

At the end of each week employees and HR will receive the reports. If the reports indicate possible signs of burnout, the company's medical service will be notified, review the case and provide whatever assistance may be necessary.

This software also features a medical appointment system, where employees can schedule or modify an appointment with the company doctor. The medical department can accept, reject and modify these appointments and will also be able to schedule an appointment for employees at risk of burnout according to the reports.

##### 3.2.8.1. Originality, contribution, and limitations of C8

The aim of this project is to create a platform for the prevention and detection of Burnout Syndrome among Telefónica employees, offering solutions where necessary. The project includes the development, testing and launch of the application over a period of 9 months, in addition to a maintenance period of 2 years.

The added-value of the proposal is that it is a personalized application; employees have access to personal reports and advice while HR is provided with group reports; comprehensive data from various sources is collected and analyzed; the application allows fluid contact with the company medical service to make appointments, etc. This is clearly an employee-centered approach to the prevention and mitigation of Burnout Syndrome.

#### 3.2.9. Contribution 9

This project is called TrusTec (34), consisting of a system able to collect, store and synthesize useful information to prevent employee fatigue. The system contains all the conventional information specialists or experts can provide about the employee, principally about mental and physical health and nutrition.

With TrusTec experts can not only diagnose the condition of employees, it also allows transparent and simple communication through a chat for greater engagement and individualization of employees.

This system gives full and native support to most sensors that collect objective parameters to identify the physical and mental health of the user, from smart watches and bracelets to body heat detection cameras. Information from user surveys or reports can be seamlessly introduced to further enrich the database.

This data collection offers interesting functionalities. By applying statistical and deep learning techniques TrusTec can display the desired parameters about the user for even more information for medical experts, individualizing queries and offering effective and simple analysis of data.

The system also makes use of cutting-edge machine learning models to identify data structures and behaviors in order to generate additional recommendations for users. These suggestions, along with those from the medical experts participating in the system will be communicated to the user, either through an app on their smart device of choice or the system website.

The user will be able to reject or accept these suggestions. If they accept, the system will create a plan along with reminders to keep users engaged, follow-up and monitoring using a simple and motivating interface to meet the user's needs.

##### 3.2.9.1. Originality, contribution, and limitations of C9

This project creates an environment for recurring information creation, which does not wait until the user feels fatigue or to take the steps themselves to deal with stress. In the event of problems in communication other employees or a temporary lack of expert personnel, the system will adapt to continue working side by side with the user, ensuring they do not suffer from exhaustion or frustration in the workplace.

The system will be set up in the branch offices of a national bank for easy and convenient access by employees, collecting employee data and generating recommendations using artificial intelligence algorithms and expert knowledge from specialists (for example, nutritionists).

The distributed software will use sensors to collect additional information the system does not incorporate by default, with a database for data storage and handling.

A web interface will also be developed and a specific interface for mobile devices for easy and fluid personal communication with employees and other company users.

#### 3.2.10. Contribution 10

The Bright Software application (35) is a multi-platform tool oriented to the care of both the physical and mental health of employees. It offers different tools to help give the user a more positive, enjoyable and, ultimately, less stressful work experience.

The aim is to demonstrate that employee productivity and employee physical and mental health are not incompatible. With a license for the product, the company will be able to acquire a KIT for each worker, consisting of a smart bracelet and sensor provided by an external supplier.

The combination of both products will make it possible to get the most out of the application since the sensor will monitor the user's body temperature, facial expression and posture, while the bracelet will monitor their heart rate.

In addition, users will also be able to purchase the application individually, without depending on their employer.

General functionalities of the app:

- Restricted access to licensed users.
- Guided meditation, breathing and stretching exercises.
- Linking the app with any wristband or smartwatch.
- Linking the app with body sensors.
- Configuration of work profiles in the app.
- Notifications or reminders will be issued if the watch or wristband detects a heart rate higher than that set by the user.
- The sensor will detect stress levels through body temperature and facial expression.

##### 3.2.10.1. Originality, contribution, and limitations of C10

This system offers the possibility to create a Mindfulness routine for users, detecting situations of stress or any other mental or physical health anomaly at work. The application asks the user to allow the company to access their data where it is evaluated, creating a personalized employee report. Thus, the client can monitor and control their stress levels while the company remains informed. The designers note the following unique features:

- The possibility to monitor mental and physical health of employees, both personally and, if necessary, by the company.
- It is possible to notify the organization of any anomalies, provided, of course, the employee agrees to share their data.
- Training exercises and routines focused on a stress reduction using mindfulness techniques.

Software will be developed to meet the needs of clients and the organization. The application can be used on any device and can be a fundamental tool for managing the wellbeing of employees.

#### 3.2.11. Contribution 11

Hubture (36) is a program which monitors levels of stress or anxiety among the employees of a company.

The first step is to create the infrastructure to which a client company will have access. Employees will be able to create their own account on the platform and thus will receive a warning when the application detects that the user is stressed or shows signs of potential burnout.

Additionally, monthly “burnout reports” will be generated with personal data of employees that will only be available to the company's medical department, and thus remaining confidential under the patient-doctor relationship. In this way, the privacy of the employee is safeguarded, avoiding any possible negative repercussions for the employee.

There will be several mechanisms to measure employee stress:

- First, employees will take a psychological test to determine their initial stress levels. This is the most basic feature of the application, which does not need to be synchronized with external sensors but with lower accuracy in measuring stress.
- Second, the company can install light intensity and temperature sensors to evaluate working conditions, ensuring these conditions are appropriate for a healthy work environment.
- Finally, wristbands and cameras can be used to measure various biometric parameters for a highly precise assessment of the user's stress levels.

##### 3.2.11.1. Originality, contribution, and limitations of C11

This project is totally unique. Currently, there is no software with these functionalities on the market. However, there are companies that deal with stress management in the workplace, such as YuCoach or CoCo Training or specialists in burnout such as the company AGS Psicólogos, but none of them offers software for monitoring and preventing workplace stress, boosting productivity while safeguarding the privacy of employees.

In addition to specific functionalities of Hubture, the designers mention other potential uses. Currently, we are witnessing a war that is having an impact around the world, causing great economic instability and high inflation in the price of food, energy and other raw materials. It has also triggered a severe social crisis, with many people seeing their lives irrevocably changed.

This situation can affect organizations and, in particular, employee productivity (both positively and negatively). Situations of uncertainty generate mental distress and instability among many, triggering high levels of stress. This presents an opportunity for this product, with many companies increasingly concerned about dealing with employee stress. On the other hand, increasing electricity costs may be a hindrance for the product, given its continuous energy use and potential clients may be reluctant to take on additional costs.

#### 3.2.12. Contribution 12

This proposal is for the Bunband software system (37), which permits the effective monitoring of employees in order to detect early symptoms of Burnout Syndrome. The system uses a smart wearable device, for example, a bracelet or wristband, that measures different parameters such as physical activity, heart rate and blood pressure.

This bracelet would be connected to the user's cell phone, with an app that collects and analyzes the data, generating useful and interpretable information in the form of graphs, alerts and weekly reports about the stress levels of the user. The main factors in stress analysis are sudden increases in blood pressure and pulse while at rest. The system also monitors the hours and quality of sleep and any alterations within a given time period to determine if the person is well or shows signs of suffering undue stress.

The app's software also has additional functionalities to take additional data into account: for example, inactivity alerts which tell the user when they have remained more than an hour without moving from their workspace. The system prompts the user to be active, even if only for a few minutes. There are also small user surveys conducted throughout the day where the user can share their mood.

Apart from biometric data and user mood, it is also important to consider the user's workload, accurately identifying the amount of work hours the user performs. This data makes it easier to identify cases where individuals are overworked, which can quickly produce unnecessary stress.

All this information is compiled in weekly and monthly reports sent directly to company superiors, enabling direct and easy communication without the need for the employee to report their situation. Thus, managers can be aware of the productivity and mood of their employees and restructure or redistribute workloads in the case excessive demands are being made of employees.

The software has the following functionalities:

- A Smartband or smart watch monitoring heart rate, blood pressure and motion. The bracelet also detects and reports sleep quality and sleep disturbance.
- An application that collects and analyzes the data from the bracelet, as well as user information provided by mood questionnaires at the end of the day and week.
- Inactivity alerts.
- A log of actual hours worked.
- Weekly summary with recommendations.
- Weekly or monthly summary sent to company superiors to assess stress level and adjust workloads.

##### 3.2.12.1. Originality, contribution, and limitations of C12

Extensive research found several proposals and studies but no current or existing projects or products with the same functionalities of our proposal. This product will be the first of its kind.

This product supposes an advance from current systems, oriented toward the work environment and using powerful and effective software connected to wearable devices.

This product encompasses a field still in its earliest stages, depending on technical capabilities currently under development. According to the designers, there is currently no exact methodology to determine what levels of stress are truly harmful; stress in minor doses has no clear negative impact. Nevertheless, extreme stress leading to Burnout Syndrome is a significant problem, especially in work environments which place special burdens or responsibilities on employees.

Studies and clinical trials in recent years on the use of portable devices to monitor stress (40, 41) have found that these devices can correctly identify stress among office employees using measurements similar to those proposed by this project. Further research is necessary to reliably determine the most indicative variables to detect stress and, especially, effective ways to combat it.

The most popular and widespread wearable devices, such as smart watches and bracelets, can measure stress by monitoring heart rate, blood pressure and accelerometers. These devices use their own applications or applications of other devices such as smartphones. Examples of these devices include Apple Watch (with Breathe or Stress Monitor apps), Samsung Galaxy Watch and Fitbit (using the Stila apps, for example).

Increasingly, companies are incorporating these devices into their workplaces (42), aware of the benefits of keeping employees informed about their own health, precisely the idea behind this proposal.

The twelve proposals presented before were evaluated based on the following criteria:

- Technical assessment of the digital transformation project. 60%−6 points out of 10.

° Innovation in the area of digital transformation.
° Quality of the proposal in terms of software development.
° Quality of the project management.
° Quality of the analysis.
° Quality of the design.

- Assessment of the creative idea in occupational safety and health. 40%−4 points out of 10.

° Appropriateness to the call for proposal.
° Originality in the area of OHS.

The following section presents a comparative analysis of the proposals.

## 4. Discussion

Several characteristics of the proposed projects were measured.

Table 2 below offers a comparative assessment of the features of the proposals.

**Table 2:** Assessment of the features of the proposals—technological solutions.

**ID**	**Short description**	**Development time**	**Development budget**	**Technology (web/app/standalone)**	**Expected use (individual/collective/both)**	**burnout identification/mitigation/both**	**Use of existing standards to determine burnout presence?**
1	Virtual personal assistant	6 months	62,579.96 €	App	Both	Both	No
2	BonusBreak	9 months	74,127.63 €	Standalone	Both	Both	No
3	Mental health monitor	12 months	115,930.00 €	App	Individual	Identification	No
4	Free time booking app	9.87 months	74,277.00 €	App/Web	Both	Both	No
5	BreakBuddies	9 months	243,232.99 €	Web	Both	Mitigation	No
6	FieldDay	1.75 months	113,015.00 €	App	Both	Mitigation	No
7	LightUp	12 months	136,368.44 €	App/standalone	Both	Mitigation	No
8	burnout WT	8.25 months	46,949.79 €	Standalone	Both	Both	Yes (BAT)
9	TrusTec	5.5 months	121,048.94 €	Standalone	Both	Both	No
10	Bright software	5.5 months	118,032.72 €	App/website	Both	Both	No
11	Hubture	12 months	100,420.00 €	App/web	Both	Both	No
12	Burnband	5.6 months	118,673.00 €	App	Individual	Both	No

The estimated costs of the projects vary widely, ranging from €46,000 to €243,000; estimated implementation times also vary widely, ranging from 5.5 to 12 months.

These differences suggest that it is important to carefully evaluate these estimates; it is essential to determine that project budgets and time frames are in line with the complexity of the project. Contributions 1, 2 and 8 propose budgets that appear to be in line with an estimated 1 year development period, providing support to identify and mitigate Burnout Syndrome with services aimed at both individuals and groups.

Interestingly, none of the projects used or integrated existing standards or tools into their project, with the exception of Contribution 8 (33), which incorporated the Burnout Assessment Tool for assessment but enhanced with the use of technology. This is important since these standards can serve as tools in measuring the effectiveness of burnout mitigation from an operational standpoint while increasing the changes of long-term return on investment in strategic terms. The MBI and BAT tools (9, 26) among others, constitute a body of academic and scientific knowledge that can lend support to the proposals and also expand the range of target companies.

Regarding the nature of the proposals themselves, 8 of the 12 projects proposed the use of Mobile Applications, 4 of the 12 explicitly mentioned using a Website-based approach while 4 of the 12 also propose a standalone approach. The feasibility and success of the proposals over the long term, and the chances of full use and integration into users' routines, depends on creating a tool which is both effective but also avoids being overly intrusive in their daily lives.

## 5. Conclusion

Given the frenetic pace of life today, it is no secret that finding a health balance between work and family responsibilities is increasing difficult. Individuals increasingly suffer from very high levels of stress, which they may not be aware of or may try to normalize rather than regarding it as a problem which must be addressed.

But advances in technology offer the opportunity to develop new and effective tools to identify, prevent and mitigate the burnout syndrome caused by stress.

However, merely relying on technology is not enough, solutions must come through creative and innovative proposals that break new ground, transcending conventional approaches. In other words, different outcomes require different and creative new approaches.

This paper describes how a creative challenge was presented to young developers who, compensating inexperience with boundless enthusiasm, were eager to develop and create impactful solutions. The challenge consisted of devising technological solutions that are effective in identifying, preventing and mitigating employee burnout.

Of the twelve proposals, four were pre-selected and one was deemed the winner of the challenge, receiving a monetary reward for effort, merit and creativity.

The variety of the proposals suggests that Burnout Syndrome can be addressed using a range of different approaches. It also suggests that much remains to be done. Some of the most important lessons learned in this exercise are:

- Both the personal and group dimensions of Burnout Syndrome must be addressed.
- It is necessary to harness the devices people already use to create bidirectional information flow: data indicators of stress (and burnout) are received and messages and advice about prevention and stress relief are sent.
- The model must be innovative in order to attract users.
- Solutions need to be attractive to both workers and employers.
- Existing tools, standards and models to address Burnout Syndrome should be incorporated; although these are more theoretical than practical, they have much to contribute to future solutions.
- There are many types of technological solutions (web applications, desktop applications, customized developments, etc.), but the most user-friendly solutions are those with real impact, reaching the largest number of people, for example web applications which are multi-user and multi-platform.
- The development time required for almost any application is estimated at 1 year; this is the minimum timeframe that proposals should consider.
- Costs vary depending on the complexity of the products or solutions.

Although all the projects were highly interesting and valuable, the wining project had the technical feasibility, budget and complexity for the greatest impact in the long term. Nevertheless, all the submitted projects described in this paper are worthy of consideration, either as basic ideas for developing future solutions, or as a starting point for brainstorming a large-scale burnout mitigation strategy.

To conclude, it is important to note that while this paper focusses on the technical aspects of the proposals, they may also offer strategic insights for the creation of a viable and effective burnout mitigation program. These may include intangible aspects such as organizational assets, management models, organizational culture, human resources and knowledge management models, business and investment strategies, etc.

## Data availability statement

The datasets presented in this study can be found in online repositories. The names of the repository/repositories and accession number(s) can be found below: https://www.promiseinnovatech.com/challengec4dx?view=article&amp;id=40:c4dx-2022-creativity-for-digital-transformation-in-organizational-health-and-safety&amp;catid=2:uncategorised.

## Author contributions

M-IS-S led the research, posing the questions underpinning the research, and managing the challenge referred to in this paper. G-LD-P worked on both the development of the justification, state of the art sections of the paper, and the summaries of the contributions. FM-D collaborated in coaching the challenge participants, providing support, and continuous feedback. AA and RM evaluated the proposals, helped determine the impact, and practical implications of this work. All authors contributed to the article and approved the submitted version.
